# Dietary Cholesterol Modulates Pathogen Blocking by *Wolbachia*

**DOI:** 10.1371/journal.ppat.1003459

**Published:** 2013-06-27

**Authors:** Eric P. Caragata, Edwige Rancès, Lauren M. Hedges, Alexander W. Gofton, Karyn N. Johnson, Scott L. O'Neill, Elizabeth A. McGraw

**Affiliations:** 1 School of Biological Sciences, Monash University, Melbourne, Victoria, Australia; 2 School of Biological Sciences, The University of Queensland, Brisbane, Queensland, Australia; 3 Institute of Molecular Biosciences, The University of Queensland, Brisbane, Queensland, Australia; Institut Pasteur, France

## Abstract

The bacterial endosymbiont *Wolbachia pipientis* protects its hosts from a range of pathogens by limiting their ability to form infections inside the insect. This “pathogen blocking” could be explained by innate immune priming by the symbiont, competition for host-derived resources between pathogens and *Wolbachia*, or the direct modification of the cell or cellular environment by *Wolbachia*. Recent comparative work in *Drosophila* and the mosquito *Aedes aegypti* has shown that an immune response is not required for pathogen blocking, implying that there must be an additional component to the mechanism. Here we have examined the involvement of cholesterol in pathogen blocking using a system of dietary manipulation in *Drosophila melanogaster* in combination with challenge by Drosophila C virus (DCV), a common fly pathogen. We observed that flies reared on cholesterol-enriched diets infected with the *Wolbachia* strains *w*MelPop and *w*MelCS exhibited reduced pathogen blocking, with viral-induced mortality occurring 2–5 days earlier than flies reared on Standard diet. This shift toward greater virulence in the presence of cholesterol also corresponded to higher viral copy numbers in the host. Interestingly, an increase in dietary cholesterol did not have an effect on *Wolbachia* density except in one case, but this did not directly affect the strength of pathogen blocking. Our results indicate that host cholesterol levels are involved with the ability of *Wolbachia*-infected flies to resist DCV infections, suggesting that cholesterol contributes to the underlying mechanism of pathogen blocking.

## Author Summary

*Wolbachia pipientis* is an intracellular bacterium that naturally infects many insect species. These bacteria can block the replication and dissemination of a variety of pathogens that coinfect the insect. In mosquitoes this effect applies to viruses including dengue and the parasite that causes malaria. This makes *Wolbachia* a promising method of controlling a number of insect-transmitted diseases of humans. Some studies suggest that pathogen blocking is due to *Wolbachia* stimulating the host innate immune system, however in the fly *Drosophila melanogaster*, blocking occurs in the absence of such an immune effect. This suggests that there is an additional component to the mechanism. Host nutrients such as cholesterol are a limiting factor in virus replication and may serve as a source of competition that underlies pathogen blocking. Here we show that competition over cholesterol contributes to the pathogen blocking effect, with higher available cholesterol levels causing less effective blocking, and increased viral titres in *Wolbachia*-infected *D. melanogaster* challenged with the pathogenic Drosophila C virus. These results suggest that competition over cholesterol is an important part of the pathogen blocking mechanism.

## Introduction

*Wolbachia* are maternally inherited bacterial endosymbionts that naturally infect an estimated 40% of all arthropod species [1]. They are primarily known for their manipulation of host reproductive biology, particularly through the phenotype of cytoplasmic incompatibility (CI), which facilitates the spread of the symbiont through wild populations [2]. Some *Wolbachia* strains manipulate their hosts in other interesting and useful ways, such as through the phenotype known as pathogen blocking, which limits the ability of many pathogenic viruses, bacteria and nematodes to grow in the host [3],[4],[5],[6],[7],[8],[9]. The phenotype has been well characterised in *Drosophila* fruit flies where it was originally discovered. Blocking occurs against many species of naturally pathogenic viruses including Drosophila C virus and Flock House virus [5],[8],[10],[11]. The effect typically involves a delay in virus-induced mortality for *Wolbachia*-infected flies, with the strength of the effect varying by strain and pathogen type [8],[10]. For some strains this is also accompanied by a delay in virus accumulation, although this is not required to delay mortality [10]. Stronger blocking occurs in strains that grow to high density, with lower density strains having little effect [10]. Together these factors imply that *Wolbachia* can cause interference with pathogen replication.

Pathogen blocking has also been well studied in mosquitoes because of their role as disease vectors. Here the effect differs from *Drosophila* in that many key vector species are naturally uninfected by *Wolbachia*, and the strength of the blocking effect is measured in terms of its impact on viral replication and transmission rather than host survival. Several naturally uninfected species, including the dengue vector *Aedes aegypti*, have been transinfected with *Wolbachia* strains from other organisms, including *w*MelPop and *w*Mel, both originally from *D. melanogaster* [12],[13]. These strains produce a strong blocking effect and inhibit the replication of dengue virus (DENV), Chikungunya virus, the filarial worm *Brugia malayi* and the model malaria parasite *Plasmodium gallinaceum* [3],[6],[7],[13]. A strong level of pathogen blocking also leads to a greatly decreased presence of DENV in mosquito saliva [3],[7],[13], which provides a means to reduce disease transmission to humans. *Wolbachia* strains that induce CI and pathogen blocking can be used to invade and replace uninfected mosquito populations [14]. The success of this strategy hinges on a strong blocking phenotype persisting in the field; consequently it is critical to determine how *Wolbachia* cause pathogen blocking.

Since its discovery, several hypotheses have emerged to explain the mechanistic basis of pathogen blocking. The first posits that the presence of the symbiont activates the insect's innate immune response, priming the host for its subsequent interaction with the vectored pathogens, [7],[6],[8],[15]. The second suggests that *Wolbachia* may outcompete pathogens for critical nutritional resources, especially given that it has a much-reduced genome and is highly dependent on the host for metabolic support [7],[16]. Given the wide range of pathogens affected by *Wolbachia* it is quite possible that a mixture of these mechanisms is acting.

The notion of “immune priming” was initially supported by heightened expression of innate immunity genes in transcriptional profiles of *A. aegypti* infected with wMelPop-CLA [7],[6]. However the *w*MelPop infection is not representative of most *Wolbachia*, over replicating to high densities and causing tissue damage in its native *D. melanogaster* [17]. Transcriptional activation of innate immunity genes could have resulted as an effect of this pathogenicity. Furthermore, as theory predicts that pathogens often have more severe effects in novel hosts [18], the infection was likely to be more virulent in the mosquito than in the fly [12]. A subsequent set of studies was then performed that compared the immune response of both the fly and the mosquito to infection with the benign (*w*Mel) and virulent (*w*MelPop) strains. The findings indicated that pathogen blocking against DENV was present in both mosquitoes and flies, but that *Drosophila* did not exhibit a clear and consistent immune response to *Wolbachia*, similar to what is seen in other infected species [9], [19],[20]. This suggests that the reported immune activation associated with *Wolbachia* infection in mosquitoes is not necessary for pathogen blocking, and while it could enhance the trait it cannot be the only mechanism operating.

An obvious alternative mechanism for pathogen blocking is competition for key cellular molecules such as cholesterol, which is critical to the biology of host, symbiont and infecting viruses. Cholesterol in insects is vital to membrane stability and cellular signalling [21],[22], and serves as the precursor to steroid hormones involved in oogenesis [23]. *Wolbachia* replication is cholesterol-dependent, requiring cholesterol-rich host membranes to form the vacuole surrounding each bacterium [24], and their survival may also be linked to host cholesterol usage [25]. Both insect and *Wolbachia* lack the biosynthetic pathways to produce cholesterol and as such both depend on and compete for dietary cholesterol. Many viruses are also dependent on host cholesterol for their replication and cellular entry [26],[27], to the extent that the immune response to some viral infections includes down regulation of sterols [28]. Consequently, manipulation of host cholesterol by *Wolbachia* could influence the ability of infecting viruses to propagate, thus producing a blocking effect.

To test whether competition for cholesterol affected *Wolbachia*-based pathogen blocking, we worked with *Drosophila melanogaster*, where blocking was first discovered [5],[8], and where methods for dietary manipulation of cholesterol are well established [29]. Using supplementation studies paired with viral infectivity assays for Drosophila C Virus (DCV), we demonstrate that viral success as measured by increased titre and faster death in flies is conferred by increased access to cholesterol in the presence of two different *Wolbach*ia strains tested. This provides the first evidence supporting a molecular competition hypothesis for *Wolbachia*-mediated pathogen blocking.

## Results

### Viral survival assays

**Figure 1:**
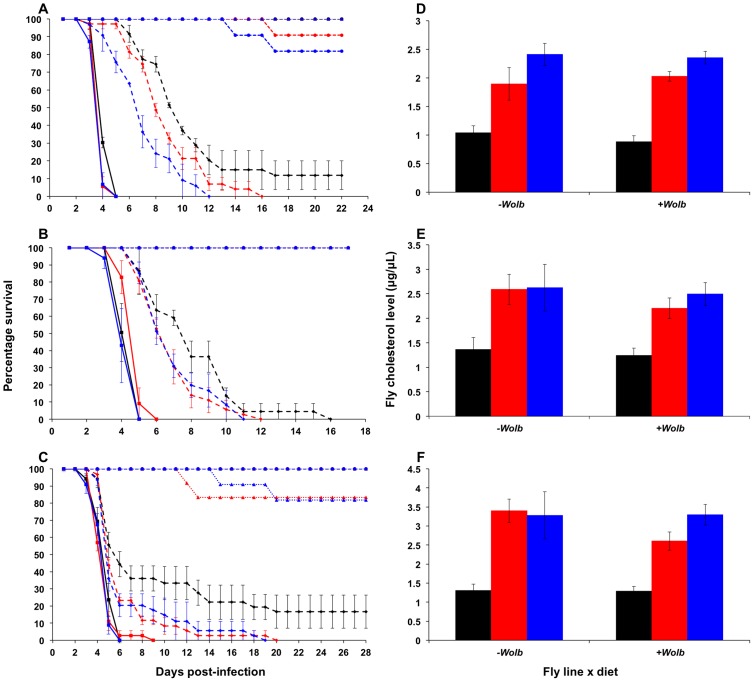
Survival curves and total cholesterol levels for ***Wolbachia*** **-infected** ***Drosophila melanogaster*** **fed cholesterol-enriched food.** Survival curves for *w*MelPop- (**A**), *w*MelCS- (**B**), and *w*Mel-infected flies (**C**) reared on cholesterol-enriched diets then challenged with DCV by injection into the haemocoel. Each curve represents one of three experiments for that strain. There was a clear effect of diet on survival, where *Wolbachia*-infected flies (dashed lines with rhomboid markers) reared on the Intermediate (red lines) and High (blue lines) cholesterol-enriched diets had a shorter average survival time than those reared on Standard food (black lines). Each line depicts the mean survival over time (± s.e.m.) for the three vials from each fly line x diet combination. Data were compared statistically using Cox Regression. A delay in virus-induced mortality was observed in *Wolbachia*-infected flies compared with uninfected (solid lines with square markers). This pathogen blocking effect occurred for all strains with the weakest blocking occurring for the *w*Mel infection. PBS-injected flies (dotted lines) experienced a high rate of survival with (circles) and without (triangles) *Wolbachia*, indicating that the death observed in DCV-infected flies was not due to trauma or buffer contamination. Mean levels (± s.e.m.) of total cholesterol and cholesteryl esters for *w*MelPop- (**D**), *w*MelCS- (**E**), and *w*Mel-infected flies (**F**) reared on Standard (black bars), Intermediate (red) and High (blue) cholesterol diets. Cholesterol quantification was performed on flies from the same bottles used in the survival assays. Data were compared statistically using univariate ANOVA followed by student's *t*-tests. Flies reared on high cholesterol diets typically had higher cholesterol levels, and this was generally associated with lower mean survival after challenge with DCV.

Upon challenge with DCV, a pathogen blocking effect was observed for all three *Wolbachia* strains, with *w*MelPop providing the greatest level of protection, *w*MelCS yielding intermediate protection, and *w*Mel the weakest. For *w*MelPop-infected flies the addition of cholesterol to dietary media reduced the protective effect of *Wolbachia* in a dose dependent manner, with increasing cholesterol concentrations in the media leading to quicker DCV-induced mortality (Cox regression – Exp_1_: *X^2^* = 14.62, *df* = 2, *P*<0.0001 (Fig. 1A); Exp_2_: *X^2^* = 11.13, *df* = 2, *P*<0.01; Exp_3_: *X^2^* = 21.61, *df* = 2, *P*<0.0001). The average survival of flies reared on the cholesterol-enriched Intermediate and High diets was approximately four and five days less, respectively, than those reared on Standard food (Table 1). On average across experiments, rearing on the Intermediate and High cholesterol diets proved a hazard that increased the likelihood of death by 2.08 and 2.80 times respectively.

**Table 1:** Mean survival times, cholesterol levels and hazard ratios for *Wolbachia*-infected flies across individual experiments.

*Wolbachia* Strain	Expt	Fly Chol. Level (µg/µL) (mean ± s.e.m.)			Survival (Days) (mean ± s.e.m.)			Expected Hazard (*B*) (95% C.I.) compared to standard diet	
		Stand	Int	High	Stand	Int	High	Int	High
***w*** **MelPop**	**1**	0.88±0.11	2.03±0.08	2.35±0.11	10.94±0.77	8.90±0.49	7.24±0.40	1.67 (1.00–2.81)	2.74 (1.61–4.65)
	**2**	1.28±0.12	2.30±0.22	2.38±0.18	15.35±1.22	10.17±1.04	10.32±0.94	2.24 (1.29–3.89)	2.13 (1.25–3.65)
	**3**	1.03±0.15	2.13±0.19	2.66±0.12	12.35±1.27	7.94±0.45	6.82±0.31	2.34 (1.38–3.97)	3.51 (2.01–6.14)
***w*** **MelCS**	**1**	1.43±0.12	NA	2.46±0.07	10.79±0.88	NA	6.71±0.32	NA	2.95 (1.66–5.23)
	**2**	1.24±0.15	2.21±0.21	2.49±0.62	10.18±0.57	7.97±0.29	8.14±0.31	2.19 (1.26–3.82)	2.07 (1.18–3.62)
	**3**	1.11±0.16	0.95±0.18	2.53±0.48	9.46±0.46	10.47±0.51	8.44±0.42	0.74 (0.45–1.21)	1.35 – (0.82–2.21)
***w*** **Mel**	**1**	1.35±0.16	3.81±0.66	2.81±0.53	6.39±0.50	5.47±0.36	7.06±0.48	1.44 (0.89–2.34)	0.86 (0.53–1.41)
	**2**	1.29±0.12	2.60±0.24	3.30±0.27	10.92±1.43	6.56±0.54	6.85±0.66	1.67 (0.98–2.79)	1.70 (1.01–2.86)
	**3**	1.26±0.28	2.52±0.23	3.49±0.15	6.18±0.56	5.79±0.29	5.68±0.27	0.99 (0.61–1.61)	1.05 (0.64–1.72)

Reduced pathogen blocking was also observed for *w*MelCS-infected flies reared on cholesterol-enriched food in two out of three experiments (Cox regression – Exp_1_: *X^2^* = 14.76, *df = *1, *P*<0.0001; Exp_2_: *X^2^* = 6.95, *df* = 2, *P*<0.05 (Fig. 1B); Exp_3_: *X^2^* = 5.64, *df* = 2, *P*>0.05). The average hazard increase across the three experiments was 1.47-fold for the Intermediate diet and 2.12-fold for the High diet. For *w*Mel-infected flies, cholesterol did not significantly affect survival (Cox Regression – Exp_1_: *X^2^* = 4.33, *df* = 2, *P*>0.05; Exp_2_: *X^2^* = 5.40, *df* = 2, *P*>0.05 (Fig. 1C); Exp_3_: *X^2^* = 0.06, *df* = 2, *P*>0.05), however in experiments two and three experiments mean survival was lower for flies from at least one of the cholesterol-enriched diets compared to those from the standard diet (Table 1). Dietary cholesterol level did not significantly affect *Wolbachia*-uninfected flies in any experiment (Figure 1). Survival curves for the experiments not depicted in Figure 1 are provided as supplementary materials (Fig. S1).

Mortality in PBS-injected lines was generally low with an average death rate of less than 5% across all injection experiments, suggesting that there was no pathogenic contamination as a result of injection (Fig. 1, Fig, S1). Flies that had their *w*MelPop infection cured by treatment with tetracycline died within seven days after infection with DCV, confirming that the pathogen blocking effect occurred due to the presence of *Wolbachia* (Fig. S2).

### Fly cholesterol levels

Cholesterol levels for flies taken from the same bottles used in each survival experiment were quantified using the Amplex Red Cholesterol Testing Kit (Invitrogen). Through statistical analysis by ANOVA, dietary cholesterol level was identified as a significant factor affecting fly cholesterol levels in all experiments (Table S1). In general, fly cholesterol levels were strongly correlated to dietary cholesterol intake (Fig. 1D–F, Fig. S2, Table S1). Fly cholesterol levels showed a strong inverse correlation to mean survival time with higher cholesterol strongly associated with increased time to death (Table 1). In four experiments (*w*MelPop Exp_2_, *w*MelCS Exp_2_, *w*Mel Exp_1_ and Exp_2_) there was no difference in cholesterol level observed between Intermediate and High diet flies, and there was also no difference in survival observed between these treatments. This suggests that even though there can be variability in cholesterol uptake between experimental replicates, the relationship between host cholesterol levels and the protective effect of pathogen blocking is strong.

### DCV accumulation

**Figure 2:**
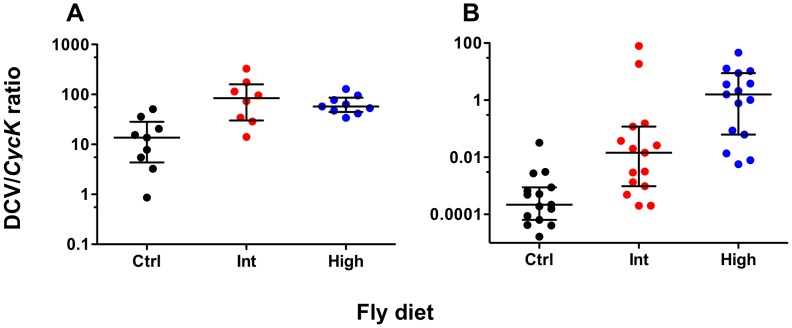
DCV levels of ***Wolbachia*** **-infected flies fed cholesterol-enriched food.** Mean normalised DCV:*CycK* expression ratios (median ± interquartile range) for *w*MelPop- (**A**) and *w*MelCS-infected flies (**B**), five days-post DCV infection. Total DCV copies were quantified using qPCR and normalised against *CycK* expression levels. Data were compared statistically using Mann-Whitney U-tests. Both strains had higher DCV levels after rearing on the Intermediate (Int) and High (High) cholesterol-enriched diets than for Standard diet (Ctrl), this suggests that it is likely that rearing on high cholesterol diets increases the rate of viral accumulation.

To determine whether increased dietary cholesterol affected the rate of viral accumulation, total DCV genome copies in pools of five *w*MelPop- and *w*MelCS-infected flies at five days post-infection were measured relative to the expression of the control gene *Cyclin K*. For both *Wolbachia* strains increased dietary cholesterol led to significantly increased viral load suggesting the presence of excess cholesterol facilitates viral propagation (Fig. 2). For *w*MelPop-infected flies DCV titre was significantly higher for the Intermediate and High cholesterol diets than for the control (Mann Whitney U-tests – Int: *U* = 8.00, *P*<0.05; High: *U = *4.00, *P*<0.01). No difference in titre was observed between the two cholesterol-enriched diets (MWU – *U* = 31.00, *P*>0.05). Median DCV:*CycK* ratio was 13.60 for the control diet, 84.38 for the Intermediate diet, and 57.22 for the High diet. DCV titre in *w*MelCS-infected flies was higher for Intermediate and High cholesterol diets (MWU – Int: *U* = 34.00, *P*<0.01, High: *U* = 3.00, *P*<0.001) and higher in the High cholesterol diet than in the Intermediate (MWU – *U* = 51.00, *P*<0.05). Median DCV:*CycK* ratio was 0.000218 for the Standard diet, 0.0145 for the Intermediate diet, and 1.590 for the High diet. Differences in accumulation between the strains reflect the use of different viral aliquots.

### *Wolbachia* density

**Figure 3:**
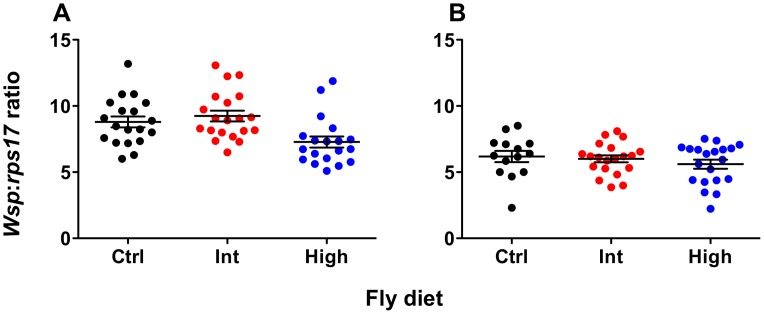
***Wolbachia*** **levels of flies reared on cholesterol-enriched food.** Mean (± s.e.m.) normalized *wsp:rps17* expression ratios for *w*MelPop- (**A**) and *w*MelCS-infected flies (**B**) during the age window that survival assays were performed. Flies were collected simultaneously with survival experiment two for *w*MelPop and survival experiment one for *w*MelCS. Total *Wolbachia* levels were quantified using qPCR. Data were compared statistically using ANOVA and student's *t*-tests corrected for multiple comparisons. For *w*MelPop-infected flies, *Wolbachia* levels were significantly lower after rearing on the High cholesterol diet, than on the Intermediate (Student's *t* test: *t = *3.370, df = 37, *P*<0.01) or Standard diets (Student's *t* test: *t = *2.586, df = 36, *P*<0.05). Interestingly, this difference in density did not have a corresponding difference in survival was between flies from the two cholesterol-enriched diets. There was no effect of diet on *Wolbachia* levels in *w*MelCS-infected flies.

*Wolbachia* density was quantified for both *w*MelPop- and *w*MelCS-infected flies using qPCR (Fig. 3). There was no effect of diet on *Wolbachia* levels for *w*MelCS-infected flies (ANOVA: *F = *0.76, *df* = 2, MSS = 1.57, *P* = 0.47). For *w*MelPop-infected flies there was a significant effect of diet on *Wolbachia* density (ANOVA: *F* = 6.24, *df* = 2, MSS = 20.42, *P = *0.0036). Here the density was significantly lower for flies from the High diet than either the Intermediate or Standard diets (Student's *t* tests – Standard-High: *t* = 2.59, *df* = 36, *P*<0.05; Int-High: *t = *3.37, *df* = 37, *P*<0.01). Interestingly, when flies from these same High and Intermediate bottles were challenged with DCV, no difference in survival was observed between the two diets (Kaplan Meier Log Rank Test: *X^2^* = 0.035, *df* = 1, *P*>0.05), indicating that this change in density did not affect pathogen blocking.

## Discussion

### Cholesterol modulates pathogen blocking by *Wolbachia*

The ability of the *Wolbachia* strains *w*MelPop and *w*MelCS to protect their host against virus appears to be dependent on the total level of host cholesterol. In *Drosophila* these *Wolbachia* strains typically protect their host against virus-induced mortality, resulting in increased survival time and delayed virus accumulation [5],[8],[10]. This protective effect was greatly reduced for flies with high levels of cholesterol, resulting in a significantly decreased survival after challenge with DCV, although survival was never compromised to the point where the protective effect was completely eliminated. Strain-specific differences in pathogen blocking strength have been previously observed and it appears there is also a strain specific difference in the effect of dietary cholesterol. In our experiments the decrease in survival time due to excess host cholesterol ranged between two and five days, with a greater survival cost observed for the *w*MelPop infection than with the *w*MelCS.

The *w*Mel strain provided reduced pathogen blocking compared to the other two strains, and consequently there was no significant effect of increased dietary cholesterol on survival time. However, in two of the three experiments involving this strain, flies from at least one of the cholesterol-enriched treatments showed increased mortality rates compared to the control, which suggests that an effect of cholesterol may have been present but was constrained by the fact that there was little increase in survival time due to *Wolbachia*. In one assay the average survival of *w*Mel-infected flies was only one day longer than uninfected flies. Stronger pathogen blocking is linked to higher *Wolbachia* densities [10],[11] and it is curious to note that of the three strains we examined *w*Mel has the lowest density [17],[30]. The effect of excess dietary cholesterol is likely linked to both *Wolbachia* density and therefore blocking strength, with strains that offer stronger blocking providing a greater delay in virus-induced mortality and potentially providing more scope for competition over cholesterol to affect viral propagation. In flies without *Wolbachia* there was no effect of cholesterol on virus-induced mortality, however it is possible that such an effect would only become apparent during infections with lower viral titres where flies would survive longer.

For all three strains the level of host cholesterol was intrinsically linked to the mean survival time, as within an experiment lines with higher cholesterol levels had lower survival times. With the exception of *w*Mel experiment 1, both significantly increased mortality and cholesterol levels were observed for flies reared on the supplemented diets compared to the control. Additionally, in cases where the two treatment diets had similar survival times, they generally had similar cholesterol levels. This suggests that there is a strong relationship between fly cholesterol levels and virus-induced mortality, and therefore represents an important determinant of pathogen blocking strength.

### Effect on virus accumulation and *Wolbachia* density

In *Drosophila*, some *Wolbachia* strains can also cause a delay in the accumulation of certain viruses, including DCV, relative to *Wolbachia*-uninfected flies [5],[10]. This effect has been attributed to the ability of *Wolbachia* to somehow interfere with the viral replication. Cholesterol has been identified as a critical factor in the replication of several viruses [26],[27],[31],[32]. In a competitive environment, if *Wolbachia* affected the usage of cholesterol by DCV, and this inhibited viral replication, we would expect to see greatly increased viral titres when cholesterol is provided in excess. For both *w*MelPop- and *w*MelCS-infected flies reared on cholesterol-enriched diets we observed a significant increase in DCV titre at five days post-infection. This suggests not only that the replication of DCV is dependent on cholesterol, but also implies a role for competition over cholesterol between *Wolbachia* and infecting viruses that can restrict viral propagation.

As an alternative to competition for cholesterol, it was possible that the observed increase in mortality and viral accumulation could have been explained if there was a corresponding decrease in *Wolbachia* density seen with the cholesterol-enriched diets. We compared the density of the *w*MelPop infection across the three host diets and observed a slight but significant decrease with the highest level of cholesterol. On the surface this might suggest that the observed effects of cholesterol on survival could have been caused by a drop in density, however there was no difference in mean survival and total cholesterol levels between intermediate and high cholesterol flies taken from the same bottles. In contrast, both lines had decreased survival and higher cholesterol than the control line, which showed no significant difference in *Wolbachia* density to the intermediate line. For *w*MelCS there was no difference in density due to diet, but mean survival and total host cholesterol differed across all three diets.

While we see that dietary supplementation with cholesterol consistently increases viral titers the effects on *Wolbachia* densities are less clear. In all but one case here, *Wolbachia* densities are unaffected by cholesterol treatment, the exception being the decline seen in *w*MelPop in response to high diet. *Wolbachia* density is likely determined by a complex series of genetic and environmental interactions [33]. There may be a threshold requirement for cholesterol beyond which increases do not lead to greater densities of the symbiont. If this were true, *Wolbachia* densities might be expected to decline under dietary restriction. The unique behavior of *w*MelPop under the high diet could be explained by an interaction between the long term rearing on the diet and the high virulence/titer associated with the strain. Regardless, the decline in *Wolbachia* densities in this treatment did not lead to corresponding decreases in blocking efficacy as measured by host survival.

### Implications for pathogen blocking

Our results further provide insight into the complex nature of *Wolbachia*-mediated pathogen blocking. This trait extends across multiple insect families, incorporating a range of effects against many different pathogens, including protection against viral-induced mortality, and blocking of pathogen replication and tissue invasion. One potential explanation for pathogen blocking in *Wolbachia*-infected mosquitoes was the broad upregulation of host immune genes associated with *Wolbachia* infection [6],[7], which has been shown to directly affect the ability of *Wolbachia* to hinder viral replication [15]. Critically, this immune priming response is not universal across *Wolbachia*-infected organisms, as *D. melanogaster* challenged with dengue virus, which is non-pathogenic in flies, show blocking of viral replication but without an accompanying immune system upregulation [9],[19]. Future work must determine whether cholesterol plays a similar role in pathogen blocking in mosquitoes, and if so whether there is also an additive relationship with the innate immune activation that has already been measured. Cholesterol supplementation of diet diminished, but did not eliminate the protective effect in our experiments, suggesting that either there is scope to completely remove blocking by further increasing cholesterol levels, or alternatively that there is a further aspect of the blocking mechanism that is as yet unidentified.

The replication of dengue and other viruses is contingent on cholesterol [32],[34], but also depends on a variety of host lipids. DENV infection perturbs lipid homeostasis and drastically alters host lipid profiles [35]. To facilitate replication DENV induces upregulation of fatty acid synthase resulting in increased fatty acid biosynthesis as well as relocation of fatty acid biosynthetic machinery to its own replication complexes [36]. *Wolbachia* is known have limited lipid biosynthesis capabilities and relies heavily on the host cell to meet this requirement [16]. As with dengue infection, *Wolbachia* induces an upregulation of fatty acid synthase in the host [19], and consequently there may be competition between *Wolbachia* and virus for key lipids beyond cholesterol that underpins pathogen blocking. Cholesterol and lipids share an association with host membranes and the Golgi apparatus, which is used by *Wolbachia* and viruses including DCV and DENV as a site for replication [24],[37],[38],[39]. Interestingly, excess cellular cholesterol accumulates around the Golgi [40], which suggests that this organelle could serve as the source of lipids for both *Wolbachia* and infecting viruses, and fuel the competitive process that contributes to pathogen blocking.

Pathogen blocking in flies has been linked to *Wolbachia* density, with high-density strains providing greater protection against virus-induced mortality [10]. The two strains where there was the greatest effect of cholesterol supplementation on pathogen blocking, *w*MelPop and *w*MelCS, both grow to high density and provide strong protection from the pathogenic effects of DCV infection, while the *w*Mel infection, which is less pervasive in fly tissues does not. This suggests that while competition for cholesterol plays an important role in pathogen blocking, the effect is stronger for strains that provide greater protection to their host, as this offers more scope for competition under conditions of high cholesterol. As for *Drosophila*, pathogen blocking in *Wolbachia*-infected *Aedes aegypti* shows a high degree of density dependence, with low-density natural infections such as those in *Aedes fluviatillis* and *Aedes albopictus* providing only minimal blocking, and transinfected infections of *w*MelPop-CLA and *w*Mel in *Aedes aegypti* growing to high density and providing strong interference against many pathogens [7],[13]. *A. aegypti*, infected with these high density strains, have been used in field releases designed to introduce *Wolbachia* into wild mosquito populations to render them incapable of dengue transmission [14]. Given our results there is scope for a role for competition over cholesterol to affect pathogen blocking in these lines.

The results presented here indicate that the mechanism of pathogen blocking is likely to be multifaceted. This in turn would suggest that development of resistance by pathogens to *Wolbachia* blocking is likely to be more difficult than if the mechanism was mediated solely through immune priming. Moreover since assays measuring the strength of pathogen blocking are conducted using laboratory animal lines reared under optimal nutritional conditions, it is possible that these assays are underestimating the strength of blocking that occurs under field conditions where insects are often subjected to extremely nutrient deficient habitats.

## Materials and Methods

### Fly rearing

*Drosophila melanogaster* were reared on one of three diets - Standard media (50 g Sugar, 17 g Torula Yeast, 15 g Agar/L of food) [41], Intermediate cholesterol or High cholesterol. Powdered cholesterol (Sigma Aldrich C3045) was dissolved in 100% ethanol to produce an increase in dietary concentration of 0.05 mg/mL for Intermediate, and 0.1 mg/mL for High. Cholesterol in ethanol was added to media during cooking after it had boiled when the temperature had decreased to approximately 60°C. 1 mL of ethanol + cholesterol was added per 40 mL of food. 1 mL of ethanol without cholesterol was added to the control diet to account for any potential effects of ethanol on fly survival. Flies were reared in bottles containing 40 mL of media at a standardised density of approximately 150 larvae per bottle. Bottles were maintained at 25°C, RH 60% in an incubator.

Three independent lines of *Drosophila melanogaster* from the w1118 background, infected with the *w*MelPop, *w*Mel and *w*MelCS *Wolbachia* strains were moved to media containing 0.3 mg/mL tetracycline-HCl [2]. After two generations females from each line were screened for the presence of the *Wolbachia* surface protein gene (*wsp*). Primers (5′ - 3′): (*wsp*F – TGGTCCAATAAGTGATGAAGAAAC); (*wsp*R – AAAAATTAAACGCTACTCCA). Flies were then screened with PCR to determine that they did not harbour a vertically transmitted DCV infection. Primers (5′ - 3′): (DCVF AGGCTGTGTTTGCGCGAAG); (DCVR – AATGGCAAGCGCACACAATTA) [42]. Once flies were free of *Wolbachia*, they were removed from tetracycline-supplemented food and transferred to bottles where uninfected male flies had been allowed to feed freely for 24 hours. This allowed for recolonisation of typical microbial infections of the digestive system that were removed with the *Wolbachia* infection by tetracycline treatment. Flies were reared on either Standard, Intermediate or High diets for between 7 and 11 generations before experiments. DCV accumulation in *w*MelCS-infected flies was measured after 30 generations on cholesterol-enriched food.

### Virus purification and titration

S2 cells were infected with DCV from previous stocks [42] by incubating for 5 days at 28°C in Schneider's standard media + 10% FBS + 1% Pen/strep. Cells were thawed and frozen to allow the release of the virus from the cells. The supernatant was centrifuged at 4000 rpm at 12°C for 25 mins and then ultracentrifuged at 25,000 rpm at 12°C for 3 hours. The pellet was resuspended in 1.5 mL 50 mM Tris pH 7.4 and left overnight at 4°C. The solution was layered using a 10–40% sucrose gradient and then ultracentrifuged again at 27,000 rpm at 12°C for 3 hours. Fractions were collected from the gradient and aliquots of the suspension were run on an SDS-PAGE gel to determine which fractions contained the bulk of the DCV. These fractions were then ultracentrifuged at 27,000 rpm at 12°C for 3 hours and the pellets resuspended in 50 mM Tris. The amount of DCV was quantified using a TCID-50 assay. S2 cells were infected with DCV in a dilution series on a 96 well cell culture plate and incubated at 28°C. After six days these cells were scored to determine which had become infected. These data were used as part of a formula that considered the dilution of virus and proportion of infected wells to determine the TCID-50 value of the aliquot in infectious units per mL [42]. Infected cells were non-confluent and surrounded by cellular debris. Three TCID-50s were performed and the overall concentration of the DCV stock was taken as the average of the three. Both TCID-50 and injection aliquots were thawed only once on the day of use.

### Survival analysis

Through experimental trials it was determined that a DCV titre of approximately 50 infectious units per fly would kill flies uninfected by *Wolbachia* in 5–7 days, and w1118-*w*Mel-infected flies in approximately 9–10 days. This concentration was selected for use in further survival assays. Male flies from each of the three diets were challenged with DCV via intra-peritoneal injection using a Nanoject II (Drummond Scientific). Flies for each injection were the same age, although age varied between 4–7 days post-eclosion between different experiments, a similar age range used in previous experiments [5]. Three vials of 12 flies each were injected per diet by infection status condition for each experiment. Additionally, one vial of flies per condition was injected with 1× PBS to serve as a control for the effects of the DCV infection. Survival was monitored daily, with deaths in the first 48 hours post-injection treated as being due to trauma. Three separate injections were conducted for each of the *w*MelPop-, *w*Mel- and *w*MelCS-infected lines. Survival curves represent a single injection experiment (Figs. 1 and S1). The effect of diet and *Wolbachia* infection on survival were analysed for each experiment using Cox Regression. Survival between treatments was then evaluated using the Kaplan-Meier log-rank test with strata = vial for each experiment separately. In principle it would be desirable to use ANOVA to test for the significance of the interaction term Wolbachia*cholesterol level on survival across all experiments, but this is not possible given the unavoidable variation in viral titer between preparations for the replicate experiments. All statistics were performed using SPSS V17 (IBM). Flies from the *w*MelPop-infected line were treated with tetracycline-hydrochloride to cure their *Wolbachia* infection. These “cured” flies were then challenged with DCV as above to determine that *Wolbachia* caused the protective effects observed in the other survival experiments.

### Cholesterol quantification

Cholesterol quantification was performed on flies from the same bottle used in each survival experiment to clarify that there was a diet-based difference in cholesterol levels between the injected lines. For each assay male flies from each line were provided only 10% sucrose for 24 hours to clear their digestive systems of cholesterol-containing media, and were then collected in pools of five. Flies were normalised by weight and then homogenized in buffer (150 mM NaCl, 50 mM Tris pH 7.5, 2 mM EDTA) to a final concentration of 10 µg/µL. Total cholesterol and cholesteryl ester levels were quantified using the Amplex Red Cholesterol Testing Kit (Invitrogen) according to the manufacturer's instructions. Data were obtained using a BioTek SYNERGYMX Fluorescent plate reader (Millennium Sciences) and compared statistically for each experiment with student's t-tests and ANOVA using Prism V 5.0d, (Graph Pad Software).

### DCV quantification

Total DCV levels were independently quantified for male *w*MelPop- and *w*MelCS-infected flies in order to determine the effects of increased dietary cholesterol on virus accumulation. Flies from the three dietary conditions were infected with DCV as in the survival experiments. Flies were collected in pools of four at 5 days post-infection. RNA was extracted using the TRIzol RNA extraction protocol (Invitrogen), and cDNA synthesised using Superscript III Reverse Transcriptase (Invitrogen). Levels of DCV were quantified relative to *Cyclin K* (*CycK* - FlyBase ID: FBgn0025674) in duplicate with a LightCycler 480 II Instrument (Roche) using LightCycler 480 SYBR Green I Master (Roche). DCV primers were as above. *CycK* primers were as previously described (5′ – 3′): (*CycK*F – GAGCATCCTTACACCTTTCTCCT); (*CycK*R – TAATCTCCGGCTCCCACTG) [43]. The qPCR profile was as previously described [42]. The expression levels of the reference and target genes were quantified in duplicate for each biological replicate. Mean Normalised DCV: *CycK* expression ratios were calculated using qGene [44]. These ratios were compared between treatments with Mann Whitney U-tests and Bonferroni-Holm multiple testing corrections using Prism V 5.0d (Graph Pad Software).

### *Wolbachia* density

*Wolbachia* density was quantified for *w*MelPop-infected flies from the same generation used in survival experiment two, and for *w*MelCS-infected flies from the same generation used in survival experiment one. DNA was extracted from 20 individual males using the ReliaPrep gDNA Tissue Miniprep System (Promega), according to manufacturer's instructions. *Wolbachia* density was then determined by relative quantitative PCR (qPCR) by comparing the abundance *wsp* to that of the single-copy *Drosophila melanogaster rps17* gene. Primers (5′ - 3′): (*RpS17*F – CACTCCCAGGTCCGTGGTAT); (*RpS17*R – GGACACTTCCGGCACGTAGT). *wsp* primers were as above. For each sample, qPCR amplification of DNA was performed in duplicate with a LightCycler 480 II Instrument (Roche) using LightCycler 480 SYBR Green I Master (Roche) according to the manufacturer's protocol. The temperature profile of the qPCR was 10 mins of pre-incubation at 95°C, 45 cycles of 95°C for 10 s, 60°C for 15 s, 72°C for 10 s. *wsp*:*rps17* ratios were obtained for each biological replicate using the LightCycler 480 II software (Roche), and then compared independently for each strain using ANOVA and student's *t*-tests with a Bonferroni multiple testing correction (Graph Pad Prism 5.0d).

## Supporting Information

Figure S1 **Survival curves and total cholesterol levels from other experiments.** Survival curves for experiments not depicted in Figure 1 – *w*MelPop experiments two (**A**) and three (**B**), *w*MelCS experiments one (**C**) and three (**D**), and *w*Mel experiments one (**E**) and three (**F**). There were noticeable differences in survival based on cholesterol content in diet for both *w*MelPop experiments, and in *w*MelCS experiment one (here there were no flies from the intermediate diet available for injection). For *w*MelCS experiment three and *w*Mel experiment one the survival effect was not necessarily related to dietary cholesterol, suggesting that there is a great deal of variability surrounding the trait. For *w*Mel experiment three there was little evidence of a pathogen blocking effect, with only a few *Wolbachia*-infected flies surviving longer than their uninfected counterparts. Figure key: *Wolbachia*-infected flies - dashed lines with rhomboid markers, uninfected flies – solid lines with square markers, PBS controls – dotted lines with circle markers, Standard diet – black line, Intermediate diet – red lines, High diet – blue lines. Mean (± s.e.m.) total cholesterol and cholesteryl ester levels for flies in the six experiments above - *w*MelPop experiments two (**G**) and three (**H**), *w*MelCS experiments one (**I**) and three (**J**), and *w*Mel experiments one (**K**) and three (**L**).
(TIF)

Figure S2 **Survival curve after** ***w*** **MelPop-cured flies were challenged with DCV.** Both *Wolbachia*-infected and –uninfected flies from all three dietary regimes were treated with tetracycline-hydrochloride for two generations to cure their *Wolbachia* infection. Upon challenge with DCV, there was no evidence of a pathogen blocking effect, with all lines showing complete mortality within seven days of infection. Figure key: *Wolbachia*-cured flies - dashed lines with rhomboid markers, uninfected flies – solid lines with square markers, PBS controls – dotted lines with circle markers, Standard diet – black line, Intermediate diet – red lines, High diet – blue lines.
(TIF)

Table S1 **ANOVA data for cholesterol quantification in each experiment.** The ANOVA comparisons for the cholesterol quantification data from all 9 experiments. In the majority of experiments, ‘Infected’ is not a statistically significant variable, suggesting that host cholesterol levels are not affected by *Wolbachia* infection. ‘Diet’ is a significant factor for all experiments, suggesting that our cholesterol-enriched diets were a critical factor affecting host cholesterol levels.
(DOCX)
