# Supplementary figures and images for: Dietary Cholesterol Modulates Pathogen Blocking by Wolbachia
Source: PLoS Pathog. 2013 Jun 27;9(6):e1003459. doi: 10.1371/journal.ppat.1003459 (PMC3694857; doi:10.1371/journal.ppat.1003459)

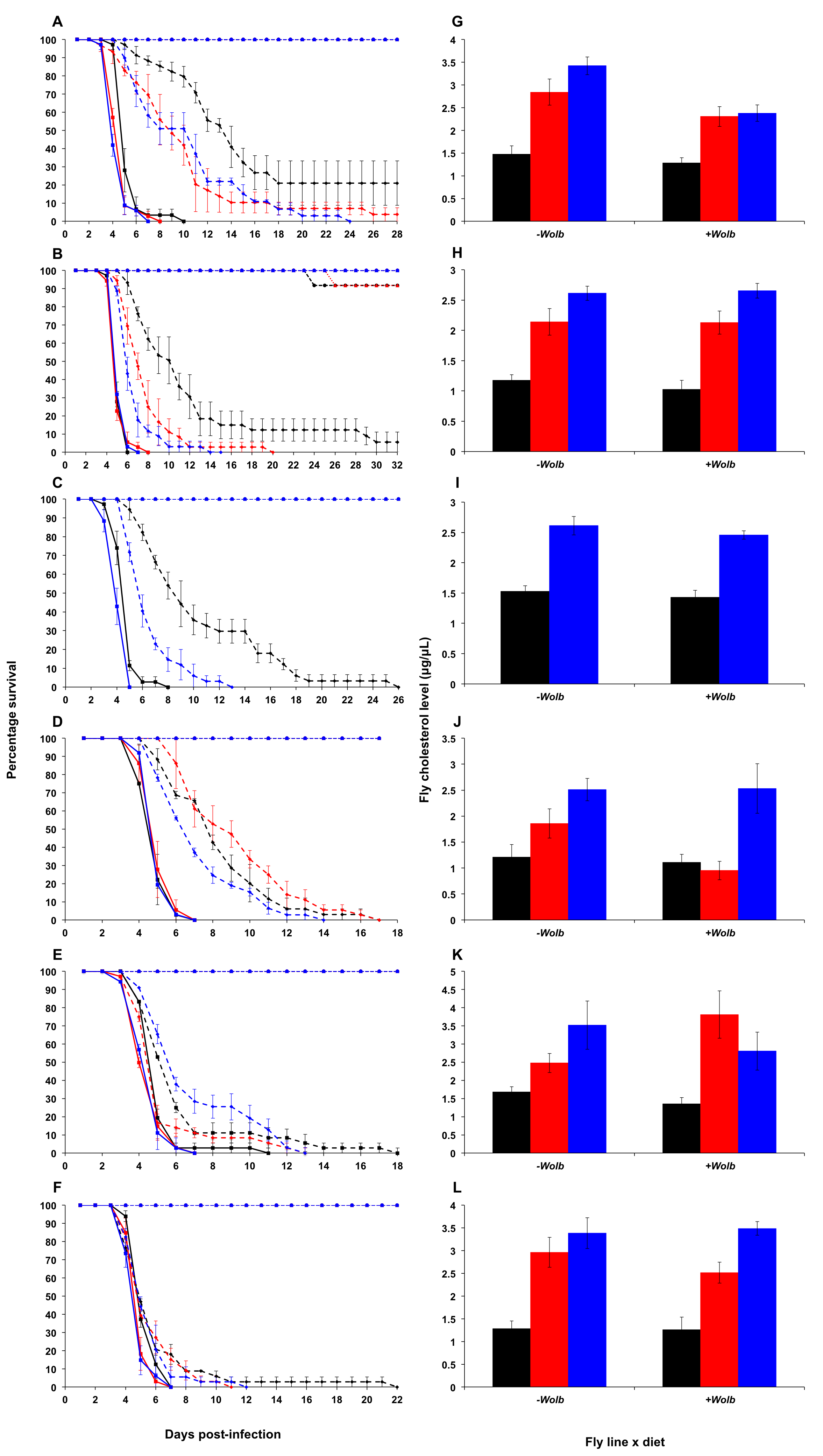

Supplement: Figure S1 — Survival curves and total cholesterol levels from other experiments. Survival curves for experiments not depicted in Figure 1 – wMelPop experiments two (A) and three (B), wMelCS experiments one (C) and three (D), and wMel experiments one (E) and three (F). There were noticeable differences in survival based on cholesterol content in diet for both wMelPop experiments, and in wMelCS experiment one (here there were no flies from the intermediate diet available for injection). For wMelCS experiment three and wMel experiment one the survival effect was not necessarily related to dietary cholesterol, suggesting that there is a great deal of variability surrounding the trait. For wMel experiment three there was little evidence of a pathogen blocking effect, with only a few Wolbachia-infected flies surviving longer than their uninfected counterparts. Figure key: Wolbachia-infected flies - dashed lines with rhomboid markers, uninfected flies – solid lines with square markers, PBS controls – dotted lines with circle markers, Standard diet – black line, Intermediate diet – red lines, High diet – blue lines. Mean (± s.e.m.) total cholesterol and cholesteryl ester levels for flies in the six experiments above - wMelPop experiments two (G) and three (H), wMelCS experiments one (I) and three (J), and wMel experiments one (K) and three (L). (TIF) [file ppat.1003459.s001.tif]

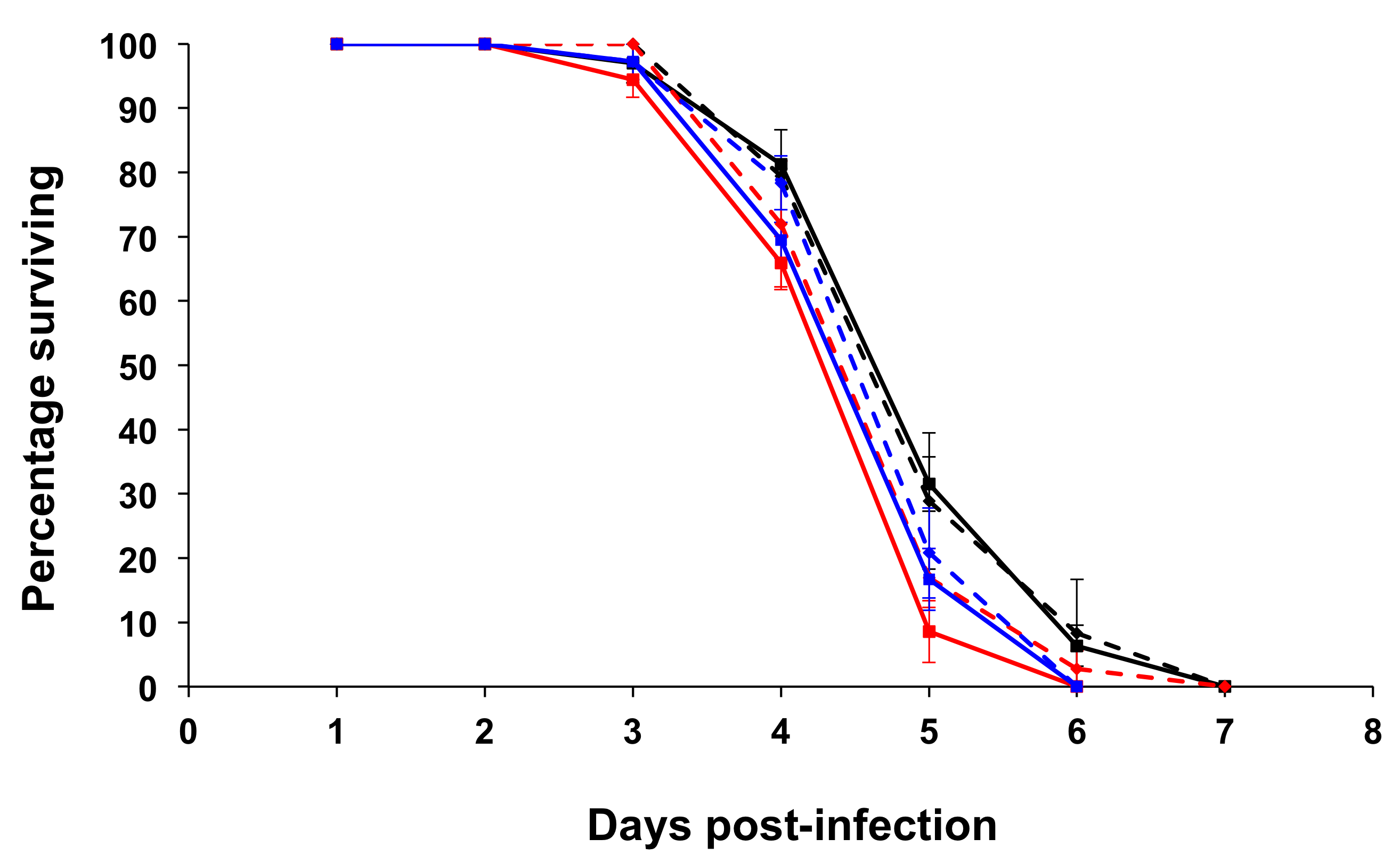

Supplement: Figure S2 — Survival curve after w MelPop-cured flies were challenged with DCV. Both Wolbachia-infected and –uninfected flies from all three dietary regimes were treated with tetracycline-hydrochloride for two generations to cure their Wolbachia infection. Upon challenge with DCV, there was no evidence of a pathogen blocking effect, with all lines showing complete mortality within seven days of infection. Figure key: Wolbachia-cured flies - dashed lines with rhomboid markers, uninfected flies – solid lines with square markers, PBS controls – dotted lines with circle markers, Standard diet – black line, Intermediate diet – red lines, High diet – blue lines. (TIF) [file ppat.1003459.s002.tif]
