# Supplementary material for: Dietary Cholesterol Modulates Pathogen Blocking by Wolbachia
Source: PLoS Pathog. 2013 Jun 27;9(6):e1003459. doi: 10.1371/journal.ppat.1003459 (PMC3694857; doi:10.1371/journal.ppat.1003459)
Supplement: Table S1 — ANOVA data for cholesterol quantification in each experiment. The ANOVA comparisons for the cholesterol quantification data from all 9 experiments. In the majority of experiments, ‘Infected’ is not a statistically significant variable, suggesting that host cholesterol levels are not affected by Wolbachia infection. ‘Diet’ is a significant factor for all experiments, suggesting that our cholesterol-enriched diets were a critical factor affecting host cholesterol levels. (DOCX) [file ppat.1003459.s003.docx]

**Table S1: ANOVA data for cholesterol quantification in each experiment**

| ***w*MelPop Exp1** | | | | | |
| --- | --- | --- | --- | --- | --- |
| Source | Type III Sum | df | Mean Square | F | Sig. |
| Corrected Model | 15.041 | 5 | 3.008 | 15.617 | <0.0001 |
| Intercept | 131.384 | 1 | 131.384 | 682.087 | <0.0001 |
| Infected | 0.008 | 1 | 0.008 | 0.044 | 0.835 |
| Diet | 14.881 | 2 | 7.441 | 38.629 | <0.0001 |
| Infected*Diet | 0.151 | 2 | 0.076 | 0.393 | 0.678 |
| Error | 6.934 | 36 | 0.193 |  |  |
| Total | 153.36 | 42 |  |  |  |
| ***w*MelPop Exp2** | | | | | |
| Source | Type III Sum | df | Mean Square | F | Sig. |
| Corrected Model | 21.584 | 5 | 4.317 | 17.858 | <0.0001 |
| Intercept | 212.603 | 1 | 212.603 | 879.548 | <0.0001 |
| Infected | 3.046 | 1 | 3.046 | 12.603 | 0.001 |
| Diet | 17.67 | 2 | 8.835 | 36.552 | <0.0001 |
| Infected*Diet | 0.867 | 2 | 0.433 | 1.793 | 0.181 |
| Error | 8.702 | 36 | 0.242 |  |  |
| Total | 242.888 | 42 |  |  |  |
| ***w*MelPop Exp3** | | | | | |
| Source | Type III Sum | df | Mean Square | F | Sig. |
| Corrected Model | 17.197 | 5 | 3.439 | 20.592 | <0.0001 |
| Intercept | 160.851 | 1 | 160.851 | 963.05 | <0.0001 |
| Infected | 0.018 | 1 | 0.018 | 0.105 | 0.748 |
| Diet | 17.111 | 2 | 8.555 | 51.223 | <0.0001 |
| Infected*Diet | 0.069 | 2 | 0.034 | 0.205 | 0.815 |
| Error | 6.013 | 36 | 0.167 |  |  |
| Total | 184.061 | 42 |  |  |  |
| ***w*MelCS Exp1** | | | | | |
| Source | Type III Sum | df | Mean Square | F | Sig. |
| Corrected Model | 7.952 | 3 | 2.651 | 30.047 | <0.0001 |
| Intercept | 112.678 | 1 | 112.678 | 1277.254 | <0.0001 |
| Infected | 0.113 | 1 | 0.113 | 1.275 | 0.27 |
| Diet | 7.834 | 1 | 7.834 | 88.805 | <0.0001 |
| Infected*Diet | 0.005 | 1 | 0.005 | 0.061 | 0.807 |
| Error | 2.117 | 24 | 0.088 |  |  |
| Total | 122.748 | 28 |  |  |  |
| ***w*MelCS Exp2** | | | | | |
| Source | Type III Sum | df | Mean Square | F | Sig. |
| Corrected Model | 13.700 | 5 | 2.74 | 4.635 | 0.002 |
| Intercept | 182.648 | 1 | 182.648 | 308.968 | <0.0001 |
| Infected | 0.465 | 1 | 0.465 | 0.787 | 0.381 |
| Diet | 13.079 | 2 | 6.54 | 11.062 | <0.0001 |
| Infected*Diet | 0.155 | 2 | 0.078 | 0.131 | 0.877 |
| Error | 21.282 | 36 | 0.591 |  |  |
| Total | 217.629 | 42 |  |  |  |
| ***w*MelCS Exp3** | | | | | |
| Source | Type III Sum | df | Mean Square | F | Sig. |
| Corrected Model | 17.661 | 5 | 3.532 | 6.477 | <0.0001 |
| Intercept | 120.611 | 1 | 120.611 | 221.171 | <0.0001 |
| Infected | 1.14 | 1 | 1.14 | 2.091 | 0.157 |
| Diet | 14.743 | 2 | 7.372 | 13.518 | <0.0001 |
| Infected*Diet | 1.777 | 2 | 0.889 | 1.63 | 0.21 |
| Error | 19.632 | 36 | 0.545 |  |  |
| Total | 157.903 | 42 |  |  |  |
| ***w*Mel Exp1** | | | | | |
| Source | Type III Sum | df | Mean Square | F | Sig. |
| Corrected Model | 33.262 | 5 | 6.652 | 4.398 | 0.003 |
| Intercept | 267.754 | 1 | 267.754 | 177.022 | <0.0001 |
| Infected | 0.1 | 1 | 0.1 | 0.066 | 0.799 |
| Diet | 24.229 | 2 | 12.114 | 8.009 | 0.001 |
| Infected*Diet | 7.316 | 2 | 3.658 | 2.418 | 0.104 |
| Error | 51.426 | 34 | 1.513 |  |  |
| Total | 358.069 | 40 |  |  |  |
| ***w*Mel Exp2** | | | | | |
| Source | Type III Sum | df | Mean Square | F | Sig. |
| Corrected Model | 34.552 | 5 | 6.91 | 9.088 | <0.0001 |
| Intercept | 268.67 | 1 | 268.67 | 353.354 | <0.0001 |
| Infected | 0.734 | 1 | 0.734 | 0.966 | 0.332 |
| Diet | 32.351 | 2 | 16.175 | 21.274 | <0.0001 |
| Infected*Diet | 1.466 | 2 | 0.733 | 0.964 | 0.391 |
| Error | 27.372 | 36 | 0.76 |  |  |
| Total | 330.594 | 42 |  |  |  |
| ***w*Mel Exp3** | | | | | |
| Source | Type III Sum | df | Mean Square | F | Sig. |
| Corrected Model | 34.870 | 5 | 6.974 | 14.82 | <0.0001 |
| Intercept | 258.89 | 1 | 258.89 | 550.135 | <0.0001 |
| Infected | 0.159 | 1 | 0.159 | 0.337 | 0.565 |
| Diet | 34.132 | 2 | 17.066 | 36.265 | <0.0001 |
| Infected*Diet | 0.579 | 2 | 0.29 | 0.616 | 0.546 |
| Error | 16.941 | 36 | 0.471 |  |  |
| Total | 310.701 | 42 |  |  |  |
